# Development of Technology for Obtaining Extracts from Powdered Herbs and Their Use in Culinary Products and Dishes

**DOI:** 10.3390/molecules31071146

**Published:** 2026-03-31

**Authors:** Gulzhan Zhumaliyeva, Urishbay Chomanov, Gulmira Kenenbay, Assem Boribay, Togzhan Zhomartkyzy

**Affiliations:** LPP Kazakh Research Institute of Processing and Food Industry, Almaty 050010, Kazakhstan; chomanov_u@mail.ru (U.C.); gkenenbay@mail.ru (G.K.); assembr@mail.ru (A.B.)

**Keywords:** plant extracts, aromatic herbs, extract production technology, infrared drying, soxhlet extraction, bioactive compounds, functional foods

## Abstract

This study aimed to determine the optimal drying, grinding, and extraction conditions for red sweet pepper, garlic, parsley, and celery to obtain concentrated extracts rich in bioactive compounds. Drying was performed using infrared ovens (FD-48 and Basic Station 3) at 30, 45, and 55 °C. The optimal temperature was 45 °C, ensuring effective moisture removal while preserving functional components. Grinding efficiency was compared between an IKA A 11 Basic analytical mill and a Pulverisette 0 vibratory micromill; the analytical mill demonstrated superior performance and processing speed. Soxhlet extraction with 96% ethanol enabled the preservation of flavor, aroma, and functional properties of the extracts. The influence of the herbal extract mixture on the organoleptic, physicochemical, and microbiological characteristics of culinary products was evaluated. For sauces, the optimal extract concentration was 5%, providing balanced taste, pleasant aroma, stable consistency, and intense color. Physicochemical analysis showed increases in protein (3.24–3.68%), ash (2.52–2.68%), dry matter (25.27–26.94%), and pH (4.11–4.24). Microbiological indicators (TAMC—3.0 × 10^2^ CFU/g; molds—21 CFU/g; yeasts—9 CFU/g) complied with regulatory standards. For meat products (meatballs and pies), the optimal extract composition (garlic 30%, red pepper 25%, parsley 25%, celery 20%) was applied at 0.3–0.7% of meat mass. Sensory evaluation identified 0.5% as optimal. The developed technology enables the production of functional food additives rich in protein, antioxidants, and flavonoids and is suitable for industrial implementation.

## 1. Introduction

Aromatic herbs are concentrated sources of biologically active compounds, including essential oils, antioxidants, phenolic compounds, and flavoring substances, extracted using various technologies. In the food industry, several extraction methods are employed, each with specific advantages and limitations. However, the production of herbal extracts in Kazakhstan remains insufficiently studied. Most of the literature focuses on general agro-industrial topics, while specialized research on herbal extract production is scarce, resulting in limited data on market trends, economic feasibility, and technical aspects. This gap complicates strategic planning and decision-making for potential investors and entrepreneurs.

There is a clear need to develop new products enriched with natural antioxidants and bioactive compounds using local plant raw materials. Implementing such research supports the use of domestic herbs in food production, contributes to scientific and technological development, and positively impacts population health and the national economy.

Herbs enhance the taste and aroma of foods and beverages without significantly contributing to caloric intake. Small quantities generally improve sensory characteristics without adverse effects [1]. Producing powdered herbal products for culinary applications presents multiple challenges, including careful analysis of raw materials, particle characteristics, and monitoring of processes under varying temperature, humidity, and storage conditions. Powders often consist of hydrophilic and thermolabile components that are sensitive to oxidation and microbial activity, making high-quality extract production complex.

Mass production of herbal concentrates and extracts facilitates the cost-effective use of herbs in catering and food industries. Early experience highlighted challenges in using certain herbs in large amounts, traditionally considered flavoring agents [2]. Modern food production increasingly relies on herbal extracts due to their aromatic and bioactive properties, emphasizing the need for efficient and gentle processing technologies that preserve valuable components.

Drying and extraction methods play a key role in maintaining bioactive compounds. Techniques such as infrared drying, spray drying, freeze-drying, ultrasound-assisted extraction, and CO_2_ extraction enable the production of concentrated plant extracts with minimal quality loss [3]. Literature reports suggest that drying at 40–50 °C is optimal for enzyme inactivation and microbiological safety while preserving thermolabile compounds, including essential oils and antioxidants [4].

This study focuses on parsley, garlic, celery, and red sweet pepper due to their high nutritional value and diverse health benefits. Parsley and celery provide vitamins C, K, and A, as well as minerals that support cardiovascular and bone health [5]. Red sweet pepper contains capsaicin with anti-inflammatory properties, while garlic is rich in allicin, effective against infections. Combining these herbs allows the creation of products with synergistic sensory and functional properties [6].

The aim of this research is to develop effective technologies for producing herbal extracts from parsley, garlic, celery, and red sweet pepper that preserve bioactive compounds, including essential oils, antioxidants, and vitamins. This study evaluates the effects of drying and grinding parameters on the quality of extracts and powders and provides insights for creating functional foods and safe, high-quality culinary additives using locally available herbs.

## 2. Results

### 2.1. Methods and Conditions

Drying is a key stage in the preparation of plant raw materials for the subsequent processing and extraction of biologically active compounds. The purpose of drying is to reduce moisture content to a level at which enzymatic activity ceases and valuable plant components are stabilized. The initial moisture content of fresh raw materials ranges from 60 to 80%, and reducing moisture to 5–12% ensures long-term storage and prevents microbial growth, in accordance with GOST 32065-2013 [7] and GOST 33271-2015 [8].

Drying was carried out at a controlled temperature of 43–45 °C in an FD-48 drying cabinet (Foshan Dalle Technology Co., Ltd., Foshan, China), which allowed uniform moisture removal without overheating and loss of biologically active compounds. The duration of the process depended on the type of raw material and its morphological characteristics: parsley and celery—17–19 h; garlic—21 h; red sweet pepper—24–30 h. The residual moisture after drying was as follows: garlic—10%; red pepper—12%; parsley—6%; celery—7%. These values are optimal for preserving the structure, color, aroma, and functional properties of the raw materials.

The nature of drying depends on the composition and type of biologically active compounds. For raw materials containing essential oils, the temperature should not exceed 30–35 °C to prevent their volatilization. Raw materials containing glycosides are dried at 50–60 °C, which rapidly halts enzymatic degradation of these compounds. Flavonoid-containing plants tolerate drying at 70–80 °C, while raw materials with ascorbic acid are dried at 80–100 °C. For most medicinal and aromatic plant materials, a temperature range of 40–50 °C is safe: enzymes are inhibited, but thermolabile compounds are preserved.

A comparative analysis showed that drying on the Basic Station at 55 °C leads to excessive moisture reduction in less than 24 h, negatively affecting product quality and functional value. Therefore, a temperature of 45 °C was chosen as optimal, ensuring minimal loss of biologically active components while meeting regulatory quality standards.

The choice of drying temperature and time directly affects the yield and preservation of biologically active compounds. Rapid moisture removal halts cell and enzyme activity, preventing degradation of thermolabile compounds. At the same time, a residual hygroscopic moisture of 8–15% does not reduce the quality of the raw material. For roots, rhizomes, and bark, the optimal yield of dry matter is 20–40%; for dried herbs, it is 35–50%; for leaves, it is 15–50%; for flowers, it is 15–20%; for fruits, it is 13–35%, depending on the juiciness and type of the plant part.

Thus, maintaining an optimal drying regime ensures effective dehydration, and the preservation of structural, organoleptic, and functional characteristics of the raw material, and creates conditions for subsequent effective extraction of biologically active compounds.

This study showed that the freeze-drying method (FD-48) and the basic drying method significantly affect the drying kinetics of the extract at different temperatures (30, 45, and 55 °C). Factorial ANOVA confirmed the presence of significant differences between the drying methods and conditions (*p* < 0.05).

The results demonstrate that with increasing temperature, the drying rate increases (residual moisture decreases). Both methods exhibit similar trends; however, freeze-drying (FD-48) provides slightly higher moisture retention during the early stages of drying.

The conducted power analysis and model assumption checks confirm the reliability of the results. Thus, the choice of drying method and temperature significantly impacts the efficiency of moisture removal from the extract and should be considered when optimizing the technological process.

### 2.2. Grinding Methods

To obtain powders from aromatic herbs, the grinding process for parsley, celery, red pepper, and garlic was studied using mechanical methods. The degree of grinding of bulk and powdered materials is one of the most important characteristics determining their technological properties.

To study and select grinding equipment for aromatic herbs, two types of mills were tested: an analytical mill (IKA brand Werke GmbH & Co. KG, Staufen, Germany) and a vibratory sieve mill (Pulverisette 0 brand, Fritsch GmbH, Idar-Oberstein, Germany). The main focus was on the milling quality, sieving efficiency, and production speed of the final product, which affected the quality of the powder.

Table 1 presents the grinding duration of aromatic herbs using two mills (the IKA analytical mill and the Pulverisette 0 vibratory sieve mill).

Based on the results presented in Table 1 and Table 2, it was shown that grinding on the vibratory sieve mill took 15–30 min, while the same process on the analytical mill lasted 3–5 min, demonstrating its significant advantage in speed.

An important characteristic of the material obtained after grinding is its particle-size distribution, which reflects the distribution of particle diameters. In this study, the particle size ranged from 3 to 5 mm. The particle size composition was determined using sieve analysis. Selecting an appropriate particle size is critical for producing high-quality products, as it directly affects the quality of the powder and, consequently, the final product. After grinding, the materials were sieved to obtain a homogeneous powder.

After 24 h of drying using the Basic Station, the residual moisture reached minimal values: garlic—2%; red pepper—0.5%; parsley—1%; celery—1%. These values exceed the GOST 33271-2015 standard [8] (5–12%) and indicate over-drying, which may lead to the loss of essential oils, vitamins, and other bioactive compounds.

Drying using the FD-48 cabinet at 45 °C achieved optimal residual moisture: garlic—10% (21 h); red pepper—12% (24 h); parsley—6% (18 h); celery—7% (21 h). These values comply with GOST 33271-2015 [8], ensuring preservation of the structure, color, aroma, and functional properties of the raw material.

Thus, the optimal drying method for aromatic herbs is 45 °C in the FD-48 drying cabinet, providing uniform dehydration without overheating and minimal losses of bioactive components.

### 2.3. Extraction Method

Extracts from aromatic herbs are concentrated products containing biologically active compounds, aromatic substances, and flavor compounds, obtained using various extraction methods. Several common methods are used in the food industry, each with its own characteristics.

Five main extraction methods are recognized: maceration, heating, Soxhlet extraction, ultrasonic extraction, and supercritical CO_2_ extraction. Each method can be described in terms of its working principle, advantages, and limitations. For example, maceration is simple and accessible but has a low yield of valuable compounds; Soxhlet extraction provides a high degree of extraction and allows process automation but requires laboratory equipment and higher energy consumption; ultrasonic extraction allows operation at low temperatures but is limited in the amount processed per cycle; supercritical CO_2_ extraction is environmentally friendly and does not use toxic solvents, but it requires expensive equipment and is complex to operate. In this study, Soxhlet extraction with 96% ethanol was chosen due to its high extraction efficiency, reproducibility, and ability to operate continuously under controlled temperature, ensuring maximal recovery of bioactive compounds.

We decided to use ethanol extracts, which provide an optimal combination of a high concentration of active compounds, product stability, and versatility for culinary applications [9,10,11,12].

For visual demonstration of the extraction process, a Soxhlet Fat 1 apparatus (BEGER Ltd. (Beger), Radomlje, Slovenia) and the obtained ethanol extracts of aromatic herbs are presented.

As a result, extracts of garlic, red sweet pepper, parsley, and celery were obtained using the Soxhlet method. This method ensures a high yield of extractive substances and allows the obtention of a concentrated product suitable for culinary use.

For an objective evaluation of the quality of the obtained extracts, organoleptic assessment of the extract mixtures was carried out, as presented in Table 2.

Based on the organoleptic evaluation, the optimal extract mixture was determined to be Mixture No. 4. It exhibits a balanced taste and aroma, moderate spiciness, and freshness, without the dominance of individual components, making it suitable for culinary applications.

A comparative analysis of extraction methods for aromatic herbs showed that the Soxhlet method is the most effective for extracting biologically active compounds. A technology for obtaining ethanol extracts from garlic, red sweet pepper, parsley, and celery was developed, including the following: raw material reception and sorting, drying, two-stage grinding, cyclic extraction, filtration, concentration, preparation of the optimal mixture, and storage.

Organoleptic evaluation of the mixtures according to GOST 31986-2012 [13] confirmed the selection of Mixture No. 4 as optimal in terms of taste and aroma. The results demonstrate the feasibility of producing concentrated functional additives while preserving organoleptic properties and biologically active compounds.

In addition, the physicochemical properties, as well as the nutritional and biological value of the selected aromatic herbs, were determined at the Food Safety Research Institute laboratory of Almaty Technological University, in the scientific laboratory for the assessment of food quality and safety. The physicochemical indicators of the aromatic herbs are presented in Table 3, while the microbiological indicators of the aromatic herb extract mixture are shown in Table 4.

The analysis of the physicochemical properties of the extract mixture showed that it has a high protein content and low moisture, ensuring stability during storage. The moderate fat and extractive substances, along with the presence of fat- and water-soluble antioxidants and flavonoids, indicate that the mixture possesses functional and nutritional value suitable for culinary applications and the development of functional food additives.

Thus, the extract mixture is characterized by a balanced physicochemical composition and high biological activity, making it suitable for use in culinary applications and for the development of functional food additives.

Microbiological analysis confirmed that the extract mixture complies with food safety requirements. Coliform bacteria and pathogenic Salmonella were not detected, while the total aerobic microbial count, molds, and yeasts were within permissible limits, demonstrating the safety and stability of the mixture during storage.

### 2.4. Effect of the Herbal Extract Mixture on Sauce Flavor

The sauce was prepared using the following procedure: the meat bones were washed and placed in a hot pan with fat. Carrots, onions, and tomato paste were then added, and all ingredients were thoroughly sautéed for 10–15 min. Water was then added to the pan in an amount just enough to cover the bones, and the dish was simmered for 50–60 min. Separately, the flour was roasted, after which the sauce and sugar were added, and the mixture was strained. The resulting samples were subjected to organoleptic evaluation.

For the preparation of red sauce with the addition of 1.5% and 10% powder mixtures, the recipe was taken according to GOST 31987-2012 [14], which applies to the dish “Basic Red Sauce”.

Table 5 presents the organoleptic characteristics of sauces with the addition of 1% herbal extract mixture.

**Figure 1:**
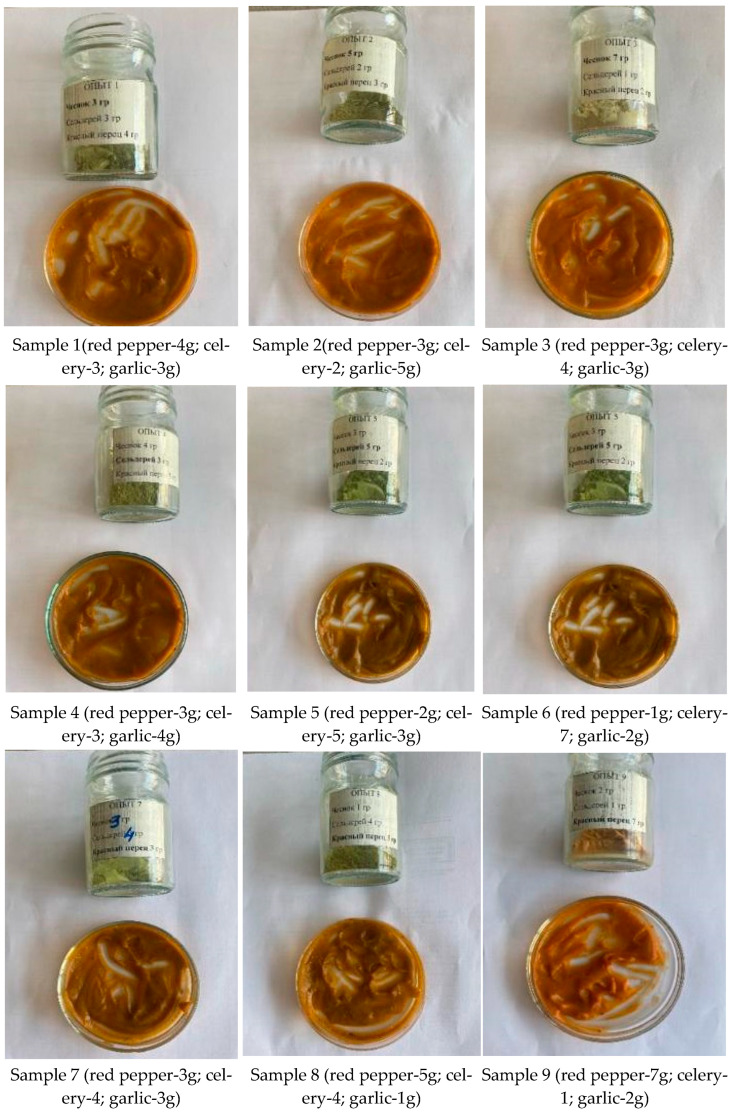
Sauces with the addition of 1% herbal extract mixture (red pepper, celery, garlic).

During the study of the organoleptic characteristics of sauces with the addition of 1% (0.2 g) herbal extracts (red pepper, garlic, celery), it was found that the color ranged from carrot to dark brown, depending on the intensity of the components. Taste analysis showed that depending on the sample, dominant notes of garlic, celery, or sweet pepper were observed, with some samples exhibiting pronounced spiciness. Aroma correlated with the primary taste characteristics, confirming the uniform distribution of aromatic compounds in the sauce. The consistency of all samples was moderately thick, indicating stable structure regardless of the composition of the herbal extracts (Figure 1).

According to the organoleptic evaluation, Sample 9 was the most pleasant in terms of taste, which may be related to its bright carrot color, the dominance of sweet pepper flavor, and the balanced aroma.

Table 6 presents the organoleptic characteristics of sauces with the addition of 5% herbal extract mixture.

**Figure 2:**
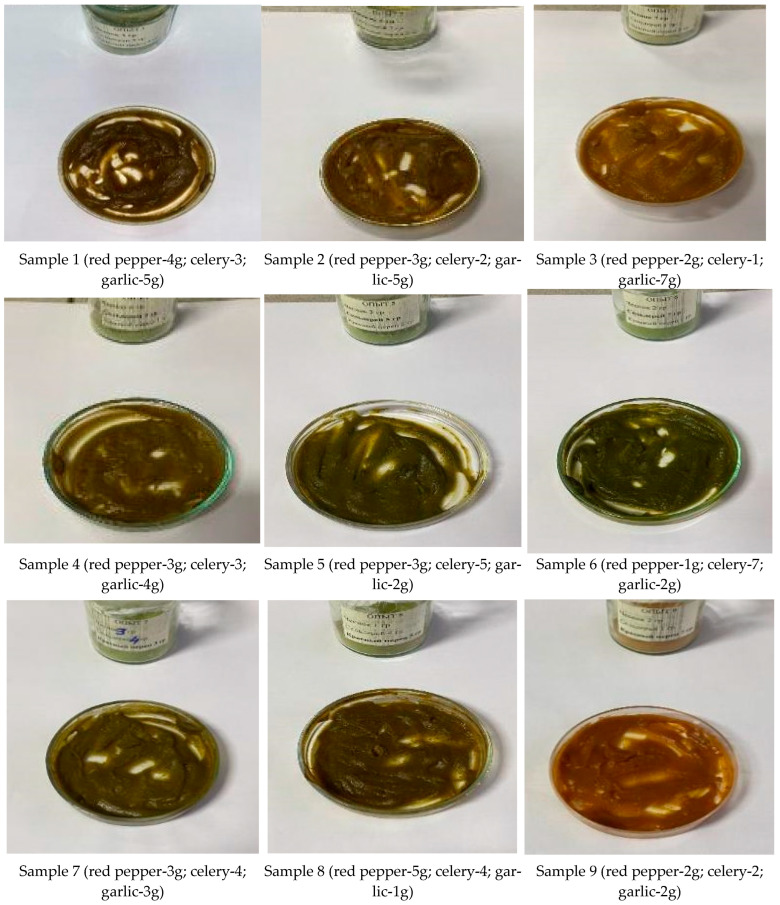
Sauces with the addition of 5% herbal extract mixture (red pepper, celery, garlic).

With the addition of 5% (1 g) of herbal extracts (red pepper, garlic, celery), the color of the sauces ranged from brown to dark green, reflecting the influence of different components on coloration. The taste characteristics varied depending on the dominant ingredient: some samples were dominated by garlic, others by celery or red pepper. Some samples exhibited noticeable spiciness. The aroma corresponded to the taste, confirming the uniform distribution of aromatic compounds. The consistency of all samples remained thick, indicating a high structural stability of the sauces (Figure 2).

Sample 9 demonstrated the most balanced taste, characterized by a carrot color, a slightly sweet flavor of red pepper, and a pleasant aroma.

Table 7 presents the organoleptic characteristics of sauces with the addition of 10% herbal extract mixture.

**Figure 3:**
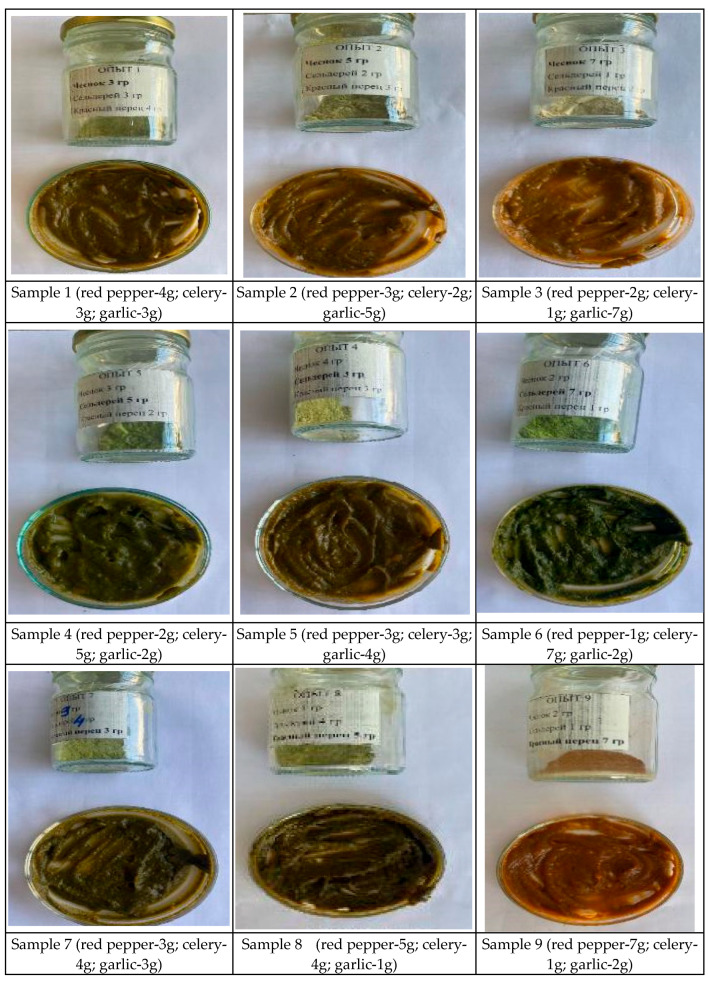
Sauces with 10% herbal extracts (red pepper, celery, garlic).

As shown in Figure 3, the analysis of organoleptic characteristics of sauces with 10% (2 g) herbal extracts (red pepper, garlic, celery) indicated that all samples had a thick consistency but differed in color, taste, and aroma.

Samples with dominant garlic flavor (1, 3, 4) exhibited dark brown shades and a pronounced garlic aroma. Samples with celery (5, 6, 7) showed greenish tones and characteristic celery aroma. Sauces with dominant red pepper (8, 9) had brighter colors and a pronounced pepper aroma.

The most pleasant in terms of taste was Sample 9, with 10% extract mixture, characterized by a carrot-like color, sweet red pepper taste, and pleasant aroma. This confirms that adding red pepper combined with a carrot base improves the organoleptic properties of the sauce.

From the experiments on adding herbal extract mixtures to sauces, the following conclusions were drawn:

- At a 1% (0.2 g) addition, the taste was barely perceptible, indicating insufficient concentration of active components to form a distinct flavor profile.
- At a 5% (1 g) addition, the taste was optimal, balanced, and pleasant, confirming the effectiveness of this concentration in improving organoleptic properties.
- At a 10% (2 g) addition, the taste became overly intense, leading to oversaturation and reduced taste quality.

To confirm the functional benefits and evaluate the effect of adding herbal extract mixtures on sauce composition, a physicochemical analysis was conducted (Table 8). Measurements were carried out according to the following standards: GOST 8756.21-89 [15], GOST 25555.4-91 [16], GOST 26186-84 [17], GOST 34570-2019 [18], GOST 31762-2012 [19], and GOST 32343-2013 [20].

The analysis of the data presented in Table 8 demonstrated that the addition of the herbal extract mixture positively influenced the physicochemical characteristics of the sauces. The protein content increased from 3.24% to 3.68%, which is attributed to the introduction of plant raw materials rich in proteins and nitrogen-containing compounds.

The ash content rose from 2.52% to 2.68%, reflecting an increase in mineral substances (sodium, potassium, magnesium, calcium, and iron) due to the incorporation of herbal extracts. The pH value increased from 4.11 to 4.24, indicating a reduction in acidity, which is typical when adding plant components containing alkaline salts.

The dry matter content increased from 25.27% to 26.94%, indicating a higher density and concentration of solid components, which also contributes to improved sauce consistency. The increases in sodium, potassium, calcium, magnesium, and iron confirm the enrichment of the product with macro- and microelements. At the same time, the fat and extractive content remained practically unchanged, indicating the preservation of nutritional value and the stability of the fat phase in the product.

Thus, the addition of 5% of the herbal extract mixture improves the nutritional value of the sauce by increasing its protein and mineral content, while also enhancing its organoleptic and physicochemical properties.

Microbiological parameters of the sauces with the added herbal extract mixture were determined in accordance with regulatory standards: GOST 10444.15-94 [21], GOST 10444.12-2013 [22], and GOST 32901-2014 [23]. The results of the microbiological analyses are presented in Table 9.

According to Table 10, the microbiological parameters of both the control and experimental samples fall within acceptable limits (GOST 32065-2013) [7]. The levels of total mesophilic aerobic and facultative anaerobic microorganisms (2.1 × 10^2^–3.0 × 10^2^ CFU/g), molds (13–21 CFU/g), and yeasts (6–9 CFU/g) do not exceed the requirements of current regulatory standards. The slight increase in microbiological counts in the experimental sample is attributed to the introduction of the plant component; however, the values remain safe and indicate the microbiological stability of the product.

The addition of the herbal extract mixture is microbiologically safe and has a comprehensive positive effect on sauce quality, making it suitable for the production of functional sauces.

Comparative analysis and sensory evaluation showed that a 5% addition of the extract mixture provides the most balanced and pleasant flavor, with optimal taste, aroma, consistency, and color, making Sample 9 the most preferable, while 1% resulted in insufficient flavor and 10% in an overly intense taste.

Physicochemical analysis confirmed that adding the herbal extract mixture enhanced the nutritional value of the sauce, increasing its protein, mineral, and dry matter content, as well as slightly raising the pH, which reflects greater product density. Microbiological parameters of the test sample remained within regulatory standards, confirming the safety and stability of the product.

The optimal dosage of the herbal extract mixture (garlic, red pepper, celery) for enhancing the organoleptic properties of sauces was determined to be 5%, which provides a balanced taste, pleasant aroma, stable consistency, and appealing color. Lower concentrations result in insufficient flavor, while higher concentrations lead to oversaturation and excessive aroma intensity. Physicochemical analysis confirmed that this addition improves the nutritional value of the sauce, increasing protein and mineral content, while microbiological parameters remain within regulatory standards, confirming the safety and stability of the product.

### 2.5. Effect of the Herbal Extract Mixture on the Flavor of Meatballs

Technologies for preparing meat pies and meatballs with the addition of an optimal herbal extract mixture (Mixture No. 4: garlic 30%, red pepper 25%, parsley 25%, celery 20%) were developed. Three dosages of the extract mixture (0.3%, 0.5%, and 0.7% of the meat mass) were tested, followed by organoleptic evaluation according to GOST 9959-2015) [24]. The results showed that the optimal concentration is 0.5%, providing the best indicators of taste, aroma, appearance, and product consistency.

The developed technologies include rationally selected recipes and a sequence of technological operations—raw material preparation, grinding, extract addition, shaping, and thermal processing—ensuring uniform distribution of active components throughout the product. The obtained data confirm the effectiveness of using herbal extracts to improve the organoleptic and functional properties of meat products and support their recommendation for industrial production.

**Figure 4:**
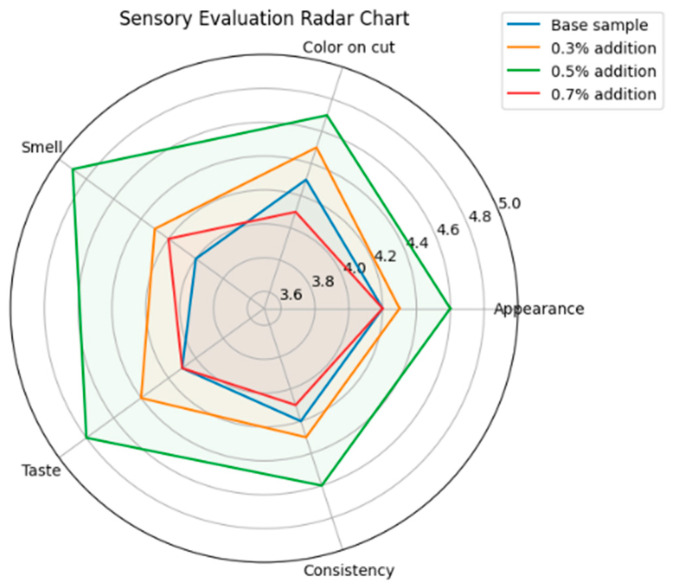
Results of the organoleptic evaluation of meatballs and meat pies with the addition of herbal extracts.

Sensory analysis results showed that the sample with a 0.5% extract mixture addition achieved the highest average score 4.80). The analysis of organoleptic evaluation is presented in Figure 4.

**Figure 5:**
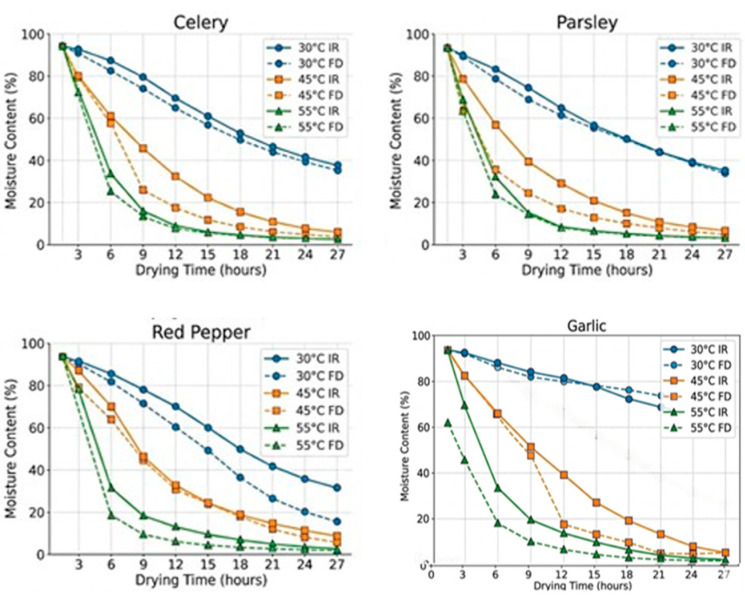
Changes in the moisture content of herbs (red pepper, parsley, garlic, celery).

Analysis of the organoleptic evaluation results showed that the addition of the herbal extract mixture improved the appearance, taste, aroma, and consistency of the products compared to the control sample. At a dosage of 0.5%, the optimal balance between aroma and taste intensity was observed, confirming the appropriateness of using this concentration in the formulations (Table 8, Figure 5).

Based on the study, a technology was developed for preparing meatballs and meat pies with the addition of herbal extracts (garlic, red pepper, parsley, celery) at 0.3–0.7% of the minced meat weight.

## 3. Discussion

The results demonstrate that processing conditions of aromatic herbs significantly influence moisture reduction kinetics and the preservation of structural and functional properties. Moisture measurements every 3 h at different temperatures indicated that the optimal drying temperature is 45 °C. Infrared drying using the Basic Station 3 equipment ensured faster moisture reduction: the moisture in red sweet pepper decreased from 93% to 25%, that in parsley decreased from 87% to 2%, that in garlic decreased from 62% to 5%, and that in celery decreased from 95% to 4% within 24 h. Drying in the FD-48 cabinet at the same temperature was slower, but final moisture levels still met regulatory requirements (red pepper 12%, parsley 0.5%, garlic 4%, celery 3%). The residual moisture values of 6–12% are within the recommended ranges for the safe storage of dry plant materials and consistent with international standards (8–15% for long-term storage and microbial control).

The literature supports the optimality of 45 °C, as moderate drying provides the best balance between efficient moisture removal and the preservation of thermolabile bioactive compounds, such as antioxidants, phenolics, and vitamins, compared to higher temperatures that accelerate oxidation and degradation [25]. At 55 °C, infrared drying caused very rapid moisture loss (red pepper 0.5%, parsley 1%), indicating excessive dehydration and the potential loss of volatile oils and other bioactive components, consistent with previous studies on drying essential-oil-containing plants [26].

Physicochemical analysis of the extract mixture confirmed that the selected drying and extraction conditions maintained a stable composition. Protein content (13.95 ± 0.13%), moderate fat and extractive substances (3.22 ± 0.007%), and low residual moisture (7.94 ± 0.07%) indicate preserved nutritional value. Antioxidant levels (water-soluble 2.15 ± 0.0334%, fat-soluble 0.51 ± 0.0063%) and flavonoids (0.68 ± 0.0081%) reflect high functional potential. Similar findings in the literature report that moderate drying preserves polyphenolic and antioxidant compounds, enhancing biological activity for food and pharmaceutical applications [27,28].

Organoleptic evaluation of the optimal extract mixture (mixture №4: garlic 30%, red pepper 25%, parsley 25%, celery 20%) showed a balanced taste and aroma, suitable for culinary use and functional foods. This aligns with the literature, showing that optimal component ratios ensure a harmonious combination of sensory and bioactive properties [28].

Microbiological analysis confirmed product safety: the absence of pathogenic Salmonella and coliforms, along with low levels of molds and yeasts, indicates stability during storage. These results comply with recommended microbial load levels for dry plant extracts and functional additives [27].

Overall, comparison with the literature confirms that the combination of optimal drying (45 °C), infrared and convective treatment, grinding, and extraction enables the production of extracts with high biological activity, optimal moisture content, storage stability, and favorable sensory properties. This validates the selected processing technology and supports its application in functional food products and feed additives with enhanced nutritional value.

## 4. Materials and Methods

### 4.1. Materials

The raw materials used in this study were aromatic herbs: red sweet pepper, garlic, celery, and parsley. These herbs were selected as sources of natural bioactive compounds, aromatic constituents, and vitamins, which enhance the taste, aroma, and nutritional properties of culinary products.

The experiments were designed to evaluate the effects of drying and grinding regimes on the quality of these materials. A full factorial design of 3 × 2 × 2 × 1 was applied, including three infrared drying temperatures (30, 45, and 55 °C), two grinding durations (3 and 5 min), two types of grinding equipment (an FD-48 drying cabinet (Foshan Dalle Technology Co., Ltd., Foshan, China) and Basic a Station 3 infrared dryer (InfraTec GmbH, Dresden, Germany)), and one extraction temperature (78 °C, corresponding to the boiling point of 96% ethanol).

Data were analyzed using analysis of variance (ANOVA) for the full factorial design, considering all main effects as well as two- and three-factor interactions. Model assumptions were verified using the Shapiro–Wilk test for normality and Levene’s test for the homogeneity of variances. Effect size was evaluated using partial η^2^, and statistically significant effects (*p* < 0.05) were further examined using Tukey’s HSD post hoc test. Statistical analyses were performed using STATISTICA 13.5 (TIBCO Software Inc., Palo Alto, CA, USA).

### 4.2. Moisture Determination

The initial moisture content of the herbs was determined gravimetrically according to GOST 32065-2013 “Dried Vegetables. General Specifications” [7]. Samples were weighed and dried at a controlled temperature of 43 °C until residual moisture reached 5–12%. Drying experiments were conducted using the FD-48 drying cabinet and the Basic Station 3 infrared dryer, ensuring precise temperature control and uniform dehydration of the samples.

Moisture loss was monitored by weighing the samples every 3 h. Optimal drying regimes were determined for each type of herb, as temperature significantly influenced the kinetics of moisture removal and the preservation of bioactive compounds.

All measurements were performed in triplicate, and results are presented as mean ± standard deviation (SD) to ensure statistical reliability. This approach allowed for the accurate evaluation of drying efficiency while minimizing degradation of thermolabile nutrients and bioactive compounds.

The changes in moisture content of the aromatic herbs (red pepper, parsley, garlic, celery) were monitored every 3 h at different drying temperatures and using different methods, outlined below.

### 4.3. Grinding

For the selection of grinding equipment for aromatic herbs, two mills were investigated: the IKA A 11 Basic analytical mill (IKA-Werke GmbH & Co. KG, Staufen, Germany) and the Pulverisette 0 vibratory micro-mill (Fritsch GmbH, Idar-Oberstein, Germany).

The Pulverisette 0 vibratory micro-mill is an ideal laboratory mill for the fine grinding of medium-hard, brittle, moist, or temperature-sensitive samples—either dry or in suspension—and for the homogenization of emulsions and pastes. It is recommended for sample preparation for RoHS testing (Restriction of Hazardous Substances). Pulverisette 0 operates by impact and friction: the pestle vibrates under an electromagnetic field, transmitting vibration to the grinding material. Coarse particles are initially broken by impact, followed by fine grinding through friction as vibrations dampen. The impact energy of the grinding ball can be adjusted to match the sample properties.

The IKA A 11 Basic analytical mill has an airtight container and batch loading, suitable for grinding hard, brittle, or inelastic materials with a hardness up to 6 on the Mohs scale. Wet materials can also be processed. Grinding is performed using the impact of tool-steel knives. Most samples are processed within 30 s per batch. High-quality milling is achieved via slow axial rotor motion. The mill includes a safety lock and indicator, which activate if the container is not fully closed or if the temperature exceeds allowable limits.

### 4.4. Extraction

Extraction was performed using a Soxhlet apparatus according to GOST 13496.15-2016 [29] with 96% ethanol [10] at ~78 °C (boiling point of ethanol) for durations specific to each plant: garlic—3 h; red pepper—2 h; parsley—6 h; celery—2.5 h. The solid-to-solvent ratio was 1:10 (mass/volume). Extracts were concentrated on a rotary evaporator under reduced pressure and stored in dark glass containers at 4 °C until further use [28].

### 4.5. Filtration and Concentration

After extraction, the mixture was filtered through ash-free filter paper to remove solid particles, ensuring extract clarity and uniformity. Extracts were concentrated on a rotary evaporator without vacuum at a water bath temperature of 75–80 °C, increasing extractive substance content, reducing solvent volume, and producing a stable product suitable for storage. Extract mixtures were prepared by combining extracts of different components to achieve a harmonious aroma.

### 4.6. Statistical Analysis

Drying kinetics of the extract mixture at 35 °C on FD-48 and Basic Station equipment were presented as mean ± standard deviation (n = 5). Error bars represent standard deviation. Different letters indicate statistically significant differences between treatments (*p* < 0.05, ANOVA with Tukey HSD post hoc comparisons).

**Figure 6:**
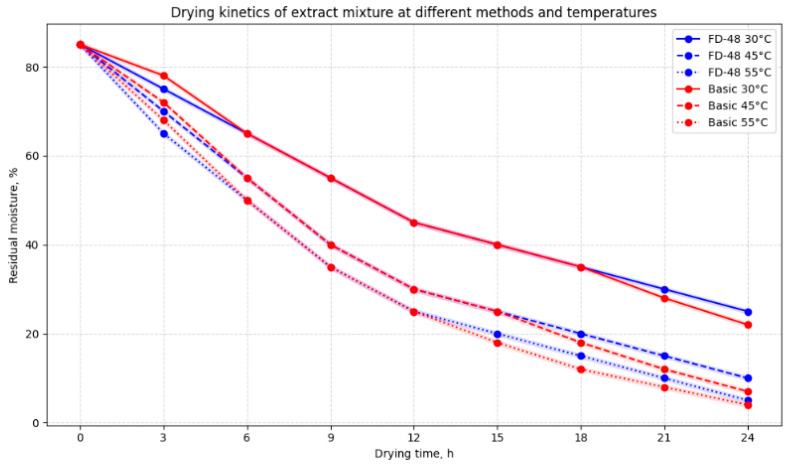
Drying kinetics of aromatic herbs (red pepper, parsley, garlic, celery) under infrared drying (Basic Station 3) and convective drying (FD-48) at 30 °C, 45 °C, and 55 °C.

A priori power analysis was performed using G*Power 3.1 at α = 0.05 and a medium effect size (f = 0.25). Each treatment was performed in five independent replicates (n = 5), providing statistical power > 80% (1 − β = 0.82) to detect main effects in factorial ANOVA. Full-factorial ANOVA included drying method, grinding time, screw rotation speed, and their interactions. Model assumptions were checked using the Shapiro–Wilk test (residual normality) and Levene’s test (variance homogeneity). Statistically significant effects (*p* < 0.05) were further analyzed with Tukey HSD multiple comparisons, and effect size was presented as partial η^2^. All statistical procedures were performed using STATISTICA 13.5 (TIBCO Software Inc., Palo Alto, CA, USA) (Figure 6).

### 4.7. Effect of the Herbal Extract Mixture on the Flavor of Sauce and Meatballs

#### 4.7.1. Effect of the Herbal Extract Mixture on the Flavor and Aroma of Red Sauce

Red sauce, characterized by a rich flavor profile and the ability to better integrate aromatic components, was used as the base. The sauce was supplemented with 1%, 5%, and 10% of the herbal extract mixture. It was found that the addition of the herbal extract mixture allowed for a more balanced taste and aroma in the red sauce. The optimal concentration of the added herbal components (1%, 5%, or 10%) was determined based on organoleptic evaluation. These results are significant for improving sauce formulations and developing new gastronomic solutions.

For the preparation of red sauce with 1%, 5%, and 10% extract mixture, the recipe followed GOST 31987-2012 [14], which applies to the dish “Basic Red Sauce” (Table 11) [25].

#### 4.7.2. Development of Formulations and Technology for Culinary Products (Meat Pies) and Dishes (Meatballs)

The composition of the herbal extract mixture recognized as optimal (Mixture No. 4—garlic 30%, red pepper 25%, parsley 25%, and celery 20%) was subsequently used to develop the technology for meat pies and meatballs. To optimize the dosage, three levels of extract addition were investigated: 0.3%, 0.5%, and 0.7% of the total weight of the minced meat. Meat pies and meatballs were prepared in accordance with the following standards (Table 12 and Table 13):

- GOST 32951-2014 [30]—Meat and meat-containing semi-finished products. General technical specifications (for meatballs and similar products);
- GOST 32589-2013 [31]—Culinary products from poultry meat. General technical specifications (for meat pies).

To assess the effect of adding the herbal extract mixture on the taste, aroma, color, and consistency of sauces and meat products, a sensory evaluation was conducted. The assessments were performed by a panel of 10 trained experts under controlled conditions. For sauces, three levels of extract addition were tested: 1%, 5%, and 10% of the total product weight, while for meat products, three levels were tested: 0.3%, 0.5%, and 0.7% of the total weight of the minced meat. Samples were evaluated using a standardized scoring system for each sensory attribute, allowing the determination of optimal preparation conditions and achieving a balanced flavor and aroma of the products.

## 5. Conclusions

As a result of the conducted experiments, the optimal processing conditions for aromatic herbs (red pepper, garlic, parsley, and celery) to obtain high-quality extracts were determined. Based on the analysis of drying kinetics and residual moisture, the optimal method was established as infrared drying at 45 °C in the FD-48 drying oven. This regime ensures uniform dehydration without overdrying and minimal loss of biologically active compounds, preserving the structure, color, and aroma of the raw materials.

The study of the grinding process showed that the IKA A 11 Basic analytical mill provides a significant advantage in terms of speed (3–5 min) and particle size distribution quality compared to the Pulverisette 0 vibratory micro-mill (15–30 min). The resulting powder is homogeneous, which is important for subsequent extraction and use in culinary products.

Soxhlet extraction with 96% ethanol allowed the obtention of concentrated extracts from all four types of herbs, retaining biologically active compounds, aroma, and flavor. Based on organoleptic evaluation, the optimal extract mixture was selected (Mixture No. 4: garlic 30%, red pepper 25%, parsley 25%, celery 20%), characterized by a balanced taste, moderate spiciness, and harmonious aroma.

Physicochemical analysis of the extract mixture revealed a high protein content (13.95 ± 0.13%), moderate levels of fat and extractive substances (3.22 ± 0.007%), low moisture (7.94 ± 0.07%), and significant concentrations of antioxidants and flavonoids, confirming the functional value of the product. Microbiological parameters met safety standards: absence of coliform bacteria and pathogenic Salmonella, and low levels of molds (16 CFU/g) and yeasts (9 CFU/g), ensuring the stability and suitability of the product for storage and culinary applications.

Thus, based on the obtained physicochemical and microbiological data, the developed technology for drying, grinding, and extracting aromatic herbs allows the production of safe, functional, and organoleptically high-quality extracts suitable for application in the food industry and the preparation of concentrated culinary additives.

## Figures and Tables

**Table 1:** Grinding parameters of aromatic herbs on different mills.

Vibratory Micromill	Red Sweet Pepper	Garlic	Parsley	Celery
Grinding on the Vibratory Micromill (Time (min))	30	30	15	15
Grinding on the Analytical Mill (Time (min))	5	5	3	3
Grinding on the Vibratory Micromill (amplitude)	2.2	2.2	2.2	2.2
Grinding on the Analytical Mill (Temperature)	40	40	25	25

**Table 2:** Organoleptic evaluation of extract mixtures.

Mixture No.	Composition of Extract Mixture (%)	Organoleptic Characteristics	Overall Evaluation
1	Garlic 50; Red Pepper 20; Parsley 15; Celery 15	Strong garlic aroma, taste too dominant	Unsatisfactory
2	Garlic 25; Red Pepper 50; Parsley 15; Celery 10	Very pungent taste, spiciness overpowers herbal aroma	Unsatisfactory
3	Garlic 20; Red Pepper 20; Parsley 30; Celery 30	Fresh aroma, mild taste, but not pronounced enough	Satisfactory
4	Garlic 30; Red Pepper 25; Parsley 25; Celery 20	Harmonious combination, moderate spiciness, balanced aroma	Optimal

**Table 3:** Physicochemical properties of the aromatic herb extract mixture.

Parameter	Extract Mixture (%)
Protein content (on a dry matter basis)	13.95 ± 0.13
Fat and extractive substances	3.22 ± 0.007
Moisture content	7.94 ± 0.07
Ash content	6.77 ± 0.04
Fat-soluble antioxidants	0.51 ± 0.0063
Water-soluble antioxidants	2.15 ± 0.0334
Flavonoids	0.68 ± 0.0081

**Table 4:** Microbiological indicators of the aromatic herb extract mixture.

Parameter	Extract Mixture
Total aerobic microbial count (CFU/g)	5.3 × 10^5^
Coliform bacteria	Not detected
Pathogenic Salmonella	Not detected
Molds	16
Yeasts	9

**Table 5:** Organoleptic characteristics of sauces containing 1% herbal extract mixture.

Sample	Color	Taste	Aroma	Consistency
1	Carrot	Garlic-dominant	Garlic-dominant	Moderately thick
2	Carrot	Spicy	Garlic-dominant	Moderately thick
3	Carrot	Spicy	Garlic-dominant	Moderately thick
4	Dark carrot	Spicy	Celery-dominant	Moderately thick
5	Light brown	Celery-dominant	Celery-dominant	Moderately thick
6	Light brown	Celery-dominant	Celery-dominant	Moderately thick
7	Dark carrot	Sweet pepper-dominant	Red pepper-dominant	Moderately thick
8	Dark brown	Sweet pepper-dominant	Red pepper-dominant	Moderately thick
9	Bright carrot	Sweet pepper-dominant	Red pepper-dominant	Moderately thick

**Table 6:** Organoleptic characteristics of sauces with 5% herbal extract mixture.

Sample	Color	Taste	Aroma	Consistency
1	Brown	Garlic-dominant	Garlic-dominant	Thick
2	Light brown	Spicy	Garlic-dominant	Thick
3	Dark carrot	Spicy	Garlic-dominant	Thick
4	Dark brown	Celery-dominant	Garlic-dominant	Thick
5	Brownish green	Celery-dominant	Garlic-dominant	Thick
6	Dark green	Celery-dominant	Celery-dominant	Thick
7	Brown	Garlic-dominant	Red pepper-dominant	Thick
8	Brown	Red pepper-dominant	Red pepper-dominant	Thick
9	Carrot	Sweet pepper	Red pepper-dominant	Thick

**Table 7:** Organoleptic characteristics of sauces with 10% herbal extract mixture.

Sample	Color	Taste	Aroma	Consistency
1	Dark brown	Dominant garlic flavor	Dominant garlic aroma	Thick
2	Brown with orange tint	Spicy	Dominant garlic aroma	Thick
3	Light brown	Strong garlic flavor	Dominant garlic aroma	Thick
4	Dark brown	Dominant garlic flavor	Dominant garlic aroma	Thick
5	Dark brown with green tint	Dominant celery flavor	Dominant garlic aroma	Thick
6	Dark green	Dominant celery flavor	Dominant garlic aroma	Thick
7	Brown with greenish tint	Dominant celery flavor	Dominant celery aroma	Thick
8	Dark brown with green tint	Dominant red pepper flavor	Dominant red pepper aroma with a hint of garlic	Thick
9	Carrot color	Sweet pepper	Dominant red pepper aroma	Thick

**Table 8:** Physicochemical characteristics of sauces with added herbal extract mixture.

Parameter	Control Sample	Experimental Sample with Sauce Addition
Protein content (on dry matter basis), %	3.24 ± 0.02	3.68 ± 0.03
Fat and extractives content, %	2.79 ± 0.04	2.75 ± 0.03
Ash content, %	2.52 ± 0.01	2.68 ± 0.02
NaCl, %	1.85 ± 0.02	1.97 ± 0.01
Nitrite, mg/100 g	6.83 ± 0.05	7.25 ± 0.05
pH	4.11 ± 0.01	4.24 ± 0.01
Dry matter, %	25.27 ± 0.26	26.94 ± 0.29
Na, mg/100 g	710.84 ± 6.95	775.18 ± 7.68
K, mg/100 g	389.75 ± 4.28	393.62 ± 4.33
Ca, mg/100 g	29.55 ± 0.32	31.03 ± 0.34
Mg, mg/100 g	17.43 ± 0.19	26.71 ± 0.38
Fe, mg/100 g	0.61 ± 0.006	0.65 ± 0.007
I, mg/100 g	0.0009 ± 0.00001	0.0012 ± 0.00001

**Table 9:** Microbiological parameters of sauces with the addition of the herbal extract mixture.

Samples	Total Mesophilic Aerobic and Facultative Anaerobic Microorganisms (CFU/g)	Molds (CFU/g)	Yeasts (CFU/g)
Control sample	2.1 × 10^2^	13	6
Experimental sample	3.0 × 10^2^	21	9

**Table 10:** Organoleptic evaluation of meat pies and meatballs with the addition of the herbal extract mixture (according to GOST 9959-2015)) [24].

Parameter	Control Sample	With 0.3% Addition	With 0.5% Addition	With 0.7% Addition
Appearance	4.5	4.6	4.8	4.4
Color in cross-section	4.4	4.5	4.7	4.3
Aroma	4.3	4.6	4.9	4.5
Taste	4.2	4.5	4.8	4.4
Consistency	4.5	4.6	4.8	4.5
Average score	4.38	4.56	4.80	4.42

**Table 11:** Recipe of the Basic Red Sauce.

Ingredient	Gross Weight (g)	Net Weight (g)
Meat bones	7500	7500
Vegetable oil	250	250
Tomato paste	600	600
Flour	500	500
Carrot	1064	798
Onion	360	302
Sugar	200	200
Herbs (celery, parsley)	190	141

**Table 12:** Recipes for meat pies with the addition of herbal extract mixture, kg (per 100 kg of unsalted raw materials).

Ingredient	Basic Recipe	With 0.3% Addition	With 0.5% Addition	With 0.7% Addition
Filling				
Beef, trimmed	150.0	150.0	150.0	150.0
Onion	20.0	20.0	20.0	20.0
Herbal extract mixture	–	0.3	0.5	0.7
Salt	1.0	1.0	1.0	1.0
Spices	to taste	to taste	to taste	to taste
Water/milk	20.0	19.7	19.5	19.3
Dough				
Wheat flour, premium grade	64.0	64.0	64.0	64.0
Sugar	4.6	4.6	4.6	4.6
Vegetable oil/margarine	6.9	6.9	6.9	6.9
Melange/egg	6.9	6.9	6.9	6.9
Salt	0.8	0.8	0.8	0.8
Fresh yeast	2.3	2.3	2.3	2.3
Water	17.0	17.0	17.0	17.0

**Table 13:** Recipes for meatballs with the addition of herbal extract mixture, kg (per 100 kg of unsalted raw materials).

Ingredient	Basic Recipe	With 0.3% Addition	With 0.5% Addition	With 0.7% Addition
Minced meat				
Beef, trimmed	100.0	100.0	100.0	100.0
Onion	15.0	15.0	15.0	15.0
Wheat bread	10.0	10.0	10.0	10.0
Milk	10.0	9.7	9.5	9.3
Egg	1.0	1.0	1.0	1.0
Herbal extract mixture	–	0.3	0.5	0.7
Salt, spices	to taste	to taste	to taste	to taste
Braising sauce				
Water	20.0	19.5	19.3	19.0
Tomato paste	5.0	5.0	5.0	5.0
Onion, sautéed	5.0	5.0	5.0	5.0
Vegetable oil	2.0	2.0	2.0	2.0
Salt, spices, bay leaf	to taste	to taste	to taste	to taste

## Data Availability

The data presented in this study are available on request from the corresponding author.

## Abbreviations

The following abbreviations are used in this manuscript:

FD-48	Infrared drying cabinet FD-48
IR	Infrared
IKA	IKA A 11 Basic analytical mill
CFU	Colony-forming unit
SD	Standard deviation
GOST	State Standard (Russia/Kazakhstan)
ANOVA	Analysis of variance
HSD	Honestly Significant Difference (Tukey test)
TAMC	Total aerobic microbial count
mL	Milliliter
min	Minute
h	Hour
%	Percent
Soxhlet	Soxhlet extraction method
temp.	Temperature
approx.	Approximately
